# Integrated Ligand-Receptor Bioinformatic and *In Vitro* Functional Analysis Identifies Active TGFA/EGFR Signaling Loop in Papillary Thyroid Carcinomas

**DOI:** 10.1371/journal.pone.0012701

**Published:** 2010-09-22

**Authors:** Debora Degl'Innocenti, Chiara Alberti, Giancarlo Castellano, Angela Greco, Claudia Miranda, Marco A. Pierotti, Ettore Seregni, Maria Grazia Borrello, Silvana Canevari, Antonella Tomassetti

**Affiliations:** Department of Experimental Oncology and Molecular Medicine, Fondazione IRCCS Istituto Nazionale dei Tumori, Milan, Italy; Louisiana State University, United States of America

## Abstract

**Background:**

Papillary thyroid carcinoma (PTCs), the most frequent thyroid cancer, is usually not life threatening, but may recur or progress to aggressive forms resistant to conventional therapies. A more detailed understanding of the signaling pathways activated in PTCs may help to identify novel therapeutic approaches against these tumors. The aim of this study is to identify signaling pathways activated in PTCs.

**Methodology/Principal Findings:**

We examined coordinated gene expression patterns of ligand/receptor (L/R) pairs using the L/R database DRLP-rev1 and five publicly available thyroid cancer datasets of gene expression on a total of 41 paired PTC/normal thyroid tissues. We identified 26 (up) and 13 (down) L/R pairs coordinately and differentially expressed. The relevance of these L/R pairs was confirmed by performing the same analysis on REarranged during Transfection (RET)/PTC1-infected thyrocytes with respect to normal thyrocytes. TGFA/EGFR emerged as one of the most tightly regulated L/R pair. Furthermore, PTC clinical samples analyzed by real-time RT-PCR expressed EGFR transcript levels similar to those of 5 normal thyroid tissues from patients with pathologies other than thyroid cancer, whereas significantly elevated levels of TGFA transcripts were only present in PTCs. Biochemical analysis of PTC cell lines demonstrated the presence of EGFR on the cell membrane and TGFA in conditioned media. Moreover, conditioned medium of the PTC cell line NIM-1 activated EGFR expressed on HeLa cells, culminating in both ERK and AKT phosphorylation. In NIM-1 cells harboring BRAF mutation, TGFA stimulated proliferation, contributing to PI3K/AKT activation independent of MEK/ERK signaling.

**Conclusions/Significance:**

We compiled a reliable list of L/R pairs associated with PTC and validated the biological role of one of the emerged L/R pair, the TGFA/EGFR, in this cancer, in vitro. These data provide a better understanding of the factors involved in the biology of PTCs and would be useful in developing combination therapeutic approaches against these cancers.

## Introduction

Thyroid cancer is the most prevalent malignancy of the endocrine system. Its incidence has increased significantly over the last several decades and it has become one of the 10 leading cancer types in females [1]. Several tumor types originate from the thyroid epithelial follicular cells and display different biological and clinical behaviors. About 80% of thyroid tumors are papillary thyroid carcinoma (PTC). Majority of the PTCs are not life threatening and are effectively treated with thyroidectomy followed by radioactive iodine ablation [2]. However, few PTCs recur or undergo progression from well-differentiated carcinoma to either poorly or undifferentiated carcinoma, and this last type is invariably fatal [3].

Many genetic alterations responsible for PTC initiation have been identified. They are mutually exclusive and include *BRAF* activating point mutations (up to 50% of cases), rearrangements of the *RET* (30%) and *NTRK1* (10%) receptor tyrosine kinase genes, and *RAS* activating mutations (present almost exclusively in the follicular variant of PTC) [4], [5]. Thus, the constitutive activation of one of the components of the RET(TRK)-RAS-BRAF-MAPK signaling pathway appears necessary for the development of PTC.

Owing to hundreds of alterations that might be merely consequential to transformation, the use of *in vitro* model of human cells expressing known oncogenes was proposed as a useful approach to focus on the pathogenic changes displayed by tumors. In this context, we have successfully applied this approach using primary human thyrocytes expressing the thyroid-specific RET/PTC1 oncogene to model PTC, and we have demonstrated that RET/PTC1 regulates the expression of a distinct set of genes involved in inflammation and tumor invasion [6].

In the past few years, the possibility to obtain global molecular profiles of tumors has provided answers to fundamental questions regarding tumor biology, as well as new tools for early detection, prognosis, and follow-up of tumors. Several groups, including ours, have determined the gene expression profile of thyroid tumors. Collectively, these studies have identified genes discriminating between benign and malignant lesions, and among the latter, specifically associated with follicular or papillary histotype [7]. With respect to PTC, specific gene expression signatures for tumors carrying RTK rearrangements or *BRAF* mutations have been detected [8], [9]. To organize and analyze such a large volume of information, new bioinformatic tools have been developed. One of these, an algorithm based on the hypothesis that two gene products participating in a common functional endpoint will have correlated transcription levels, was designed for detecting dysregulation of autocrine/paracrine ligand/receptor (L/R) signaling loops [10]. On the basis of this algorithm, we implemented the previously used L/R database [10], naming it DRLP-rev1 [11], and by a systematic meta-analysis of publicly available epithelial ovarian cancer expression array datasets we gave in this pathology the proof-of-principle of the statistical and biological validity of the correlation of the L/R gene expression patterns [11].

Here, we examined the gene expression patterns of L/R with respect to their possible role as signaling pathways activated in PTC. Using the DRLP-rev1 database to carry out meta-analysis of five publicly available datasets that reported gene expression data in thyroid cancer and normal thyroid tissues from the same patients, we identified 26 (up) and 13 (down) L/R pairs that were regulated in PTCs relative to their normal tissue counterparts. We then compared these L/R pairs with those differentially expressed in RET/PTC1-infected thyrocytes with respect to normal thyrocytes. Both the analyses identified the coordinated expression of the TGFA/EGFR L/R pair in tumor samples when compared with normal tissue. To validate this in silico analysis, functional experiments in thyroid cancer cell lines demonstrated the presence of a functional TGFA/EGFR autocrine signaling loop, which sustained the proliferation of PTC cells and contributed to the activation of the PI3K/AKT pathway independent of MEK/ERK pathway.

## Results

### Analysis of L/R pairs differentially expressed between normal and tumor thyroid samples from PTC patients

To identify autocrine/paracrine loops of signaling activated in transformed vs. normal thyroid cells, we analyzed 5 datasets (Table 1), representing 41 patients, in which the gene expression profiles of thyroid carcinoma samples were compared with those from contralateral normal thyroid. First, the DLRP-rev1 gene list was used to extract all the L/R pairs significantly correlated in normal controlateral or in PTC samples Subsequently, a paired *t*-test was used to identify significantly regulated Ligands and Receptors in PTCs, relative to their normal counterparts (see Materials and Methods for criteria of selection). By this analysis 26 and 13 L/R pairs were found up- and down-regulated, respectively, in PTCs relative to their normal counterparts (Table 2). Among the L/R pairs upregulated in tumor samples were genes involved in: chemokine pathways (CCL13/CCR1, 2 datasets), growth pathways (INHBB/ACVR1, 4 datasets; TGFA/EGFR, 2 datasets; TNC/ANXA2, 4 datasets), and motility and adhesion (PLAU/PLAUR, 2 datasets; SEMA3F/NRP2, 4 data sets; SPP1/ITGAV, 3 datasets; SPP1/CD44, 3 datasets; SPP1/ITGA9, 2 datasets).

**Table 1:** Characteristics of the explored datasets.

Code	Ref.	Year	Platform	Probesets/Clones	PTC	Normal contralateral
**I**	Vasko V [37]	2007	U133Plus 2.0	47000	7	4
**II**	GSE3678*	2006	U133 Plus 2.0	47000	7	7
**III**	Jarzab B [38]	2005	Oligo (U133A)	22283	23	16
**IV**	Aldred MA [39]	2004	Oligo (U95Av2)	12626	6	6
**V**	Huang Y [40]	2001	Oligo (U95Av2)	12626	8	8

*For this dataset, a Geo accession number is given.

**Table 2:** Ligand (L) or Receptor (R) genes modulated in PTCs with respect to normal contralateral tissues^1^.

	Dataset (no. of samples)	I (4)		II (7)		III (16)		IV (6)		V (8)	
L/R	Gene Symbol	T/N	*P-value*	T/N	*P-value*	T/N	*P-value*	T/N	*P-value*	T/N	*P-value*
			(FDR)		(FDR)		(FDR)		(FDR)		(FDR)
**Angiogenesis**											
**L**	**ANGPTL1**	**0.20**	*0.0002*	**0.21**	*0.0010*						
			(0.0177)		(0.0221)						
R	TEK	**0.70**	*0.0338*	**0.63**	*0.0064*	**0.63**	*0.0000*				
			(0.2251)		(0.0605)		(0.0000)				
**L**	**VEGF**	**0.60**	*0.0045*	**0.77**	*0.0005*	**0.56**	*0.0033*				
			(0.0799)		(0.0176)		(0.0088)				
R	FLT1			**0.53**	*0.0009*	**0.77**	*0.0218*				
					(0.0206)		(0.0410				
R	KDR			**0.59**	*0.0132*	**0.71**	*0.0208*				
					(0.0984)		(0.0396)				
R	NRP1			**0.77**	*0.0064*						
					(0.0605)						
**Cytokines**											
**L**	**IFNA4**			**1.25**	*0.0467*						
					(0.2013)						
R	IFNAR2			**1.43**	*0.0161*	**1.43**	*0.0006*	**1.25**	*0.0097*		
					(0.1074)		(0.0020)		(0.0362)		
**L**	**IFNA7**			**1.25**	*0.0044*						
					(0.0507)						
R	IFNAR2			**1.43**	*0.0161*	**1.43**	*0.0006*	**1.25**	*0.0097*		
					(0.1074)		(0.0020)		(0.0362)		
**L**	**TNFSF10**	**0.40**	*0.0007*			**0.50**	*0.0004*	**0.77**	*0.0453*		
			(0.0265)				(0.0014)		(0.1096)		
R	TNFRSF11B	**0.10**	*0.0000*	0.19	*0.0001*	**0.18**	*0.0000*				
			(0.0073)		(0.0079)		(0.0000)				
**Chemokines**											
**L**	**CCL13**	**1.79**	*0.0292*	**3.33**	*0.0004*	**5.00**	*0.0000*				
			(0.2031)		(0.0138)		(0.0000)				
R	CCR3			**1.11**	*0.0465*						
					(0.2013)						
R	CCR1			**2.00**	*0.0147*	**2.00**	*0.0002*				
					(0.1016)		(0.0007)				
**L**	**CXCL12**	**0.20**	*0.0003*	**0.45**	*0.0054*	**0.29**	*0.0000*	**0.42**	*0.0005*		
			(0.0216)		(0.0529)		(0.0003)		(0.0047)		
R	CXCR4	**0.40**	*0.0124*								
			(0.1291)								
**Growth factors**											
**L**	**AREG**					**2.00**	*0.0163*				
							(0.0322)				
R	EGFR			**1.67**	*0.0122*	**1.67**	*0.0055*	**1.25**	*0.0217*		
					(0.0937)		(0.0134)		(0.0609)		
**L**	**BMP7**			**1.25**	*0.0030*						
					(0.0424)						
R	ACVR1			**1.67**	*0.0275*	**1.43**	*0.0001*	**2.00**	*0.0002*	**1.67**	*0.0003*
					(0.1474)		(0.0004)		(0.0036)		(0.358)
**L**	**BTC**			**1.25**	*0.0310*						
					(0.1577)						
R	EGFR			**1.67**	*0.0122*	**1.67**	*0.0055*	**1.25**	*0.0217*		
					(0.0937)		(0.0134)		(0.0609)		
R	ERBB3	**3.70**	*0.0011*	**3.33**	*0.0000*	**2.00**	*0.0003*	**1.43**	*0.0210*	**2.00**	*0.0039*
			(0.0373)		(0.0006)		(0.0012)		(0.0609)		(0.0871)
**L**	**DTR**					**2.50**	*0.0008*				
							(0.0027)				
R	EGFR			**1.67**	*0.0122*	**1.67**	*0.0055*	**1.25**	*0.0217*		
					(0.0937)		(0.0134)		(0.0609)		
**L**	**INHBA**					**5.00**	*0.0000*				
							(0.0000)				
R	ACVR1			**1.67**	*0.0275*	**1.43**	*0.0001*	**2.00**	*0.0002*	**1.67**	*0.0003*
					(0.1474)		(0.0004)		(0.0036)		(0.0358)
**L**	**INHBB**	**2.38**	*0.0080*	**2.00**	*0.0112*	**2.00**	*0.0000*	**3.33**	*0.0000*	**2.50**	*0.0000*
			(0.1021)		(0.0900)		(0.0001)		(0.0000)		(0.0194)
R	ACVR1			**1.67**	*0.0275*	**1.43**	*0.0001*	**2.00**	*0.0002*	**1.67**	*0.0003*
					(0.1474)		(0.0004)		(0.0036)		(0.0358)
**L**	**JAG2**	**1.79**	*0.0070*	**1.25**	*0.0050*	**1.43**	*0.0010*				
			*(*0.0961)		*(*0.0529)		*(*0.0033)				
R	NOTCH2			**1.25**	*0.0416*						
					(0.1895)						
R	NOTCH3	**1.69**	*0.0140*								
			(0.1402)								
**L**	**TGFA**	**4.17**	*0.0001*	**3.33**	*0.0000*	**5.00**	*<1e-07*				
			(0.0073)		(0.0006)		(<1e-07)				
R	EGFR			**1.67**	*0.0122*	**1.67**	*0.0055*	**1.25**	*0.0217*		
					(0.0937)		(0.0134)		(0.0609)		
**L**	**TGFB1**	**2.38**	*0.0197*	**1.25**	*0.0192*	**2.00**	*0.0000*			**1.11**	*0.0195*
			(0.1652)		(0.1190)		(0.0001)				(0.2025)
R	TGFBR1			**1.43**	*0.0050*						
					(0.0529)						
**L**	**TNC**			**2.50**	*0.0262*	**3.33**	*0.0004*	**2.00**	*0.0101*	**2.50**	*0.0188*
					(0.1453)		(0.0014)		(0.0362)		(0.1996)
R	ANXA2			**1.43**	*0.0151*	**1.67**	*0.0000*	**1.67**	*0.0007*	**1.43**	*0.0076*
					(0.1024)		(0.000)		(0.0050)		(0.1227)
**L**	**BMP2**	**0.20**	*0.0089*	**0.23**	*0.0002*	**0.45**	*0.0003*				
			(0.1105)		(0.0118)		(0.0010)				
R	BMPR1A	**0.50**	*0.0035*	**0.71**	*0.0033*	**0.53**	*0.0000*				
			(0.0725)		(0.0448)		(0.0000)				
**L**	**BMP7**	**0.40**	*0.0106*	**0.71**	*0.0371*	**0.59**	*0.0022*				
			(0.1244)		(0.1819)		(0.0063)				
R	BMPR1A	**0.50**	*0.0035*	**0.71**	*0.0033*	**0.53**	*0.0000*				
			(0.0725)		(0.0448)		(0.0000)				
**L**	**FGF7**					**0.63**	*0.0002*				
							(0.0008)				
R	FGFR2	**0.40**	*0.0041*	**0.43**	*0.0008*	**0.26**	*0.0000*	**0.83**	*0.0032*		
			(0.796)		(0.0206)		(0.0000)		(0.0149)		
**L**	**FGF11**			**0.71**	*0.0095*						
					(0.0803)						
R	FGFR2	**0.40**	*0.0041*	**0.43**	*0.0008*	**0.26**	*0.0000*	**0.83**	*0.0032*		
			(0.796)		(0.0206)		(0.0000)		(0.0149)		
**L**	**FGF13**			**0.53**	*0.0390*	**0.48**	*0.0005*				
					(0.1841)		(0.0018)				
R	FGFR2	**0.40**	*0.0041*	**0.43**	*0.0008*	**0.26**	*0.0000*	**0.83**	*0.0032*		
			(0.796)		(0.0206)		(0.0000)		(0.0149)		
**L**	**PTN**	**0.40**	*0.0015*	**0.53**	*0.0033*	**0.45**	*0.0006*				
			(0.0503)		(0.0448)		(0.0020)				
R	PTPRB			**0.63**	*0.0001*						
					(0.0079)						
**L**	**TGFB1**	**2.38**	*0.0197*	**1.25**	*0.0192*	**2.00**	*0.0000*			**1.11**	*0.0195*
			(0.1652)		(0.1190)		(0.0001)				(0.2025)
R	TGFBR2	**0.50**	*0.0241*	**0.63**	*0.0005*	**0.67**	*0.0003*				
			(0.1842)		(0.0176)		(0.0010)				
R	TGFBR3	**0.40**	*0.0039*	**0.45**	*0.0004*	**0.45**	*0.0002*				
			(0.0778)		(0.0138)		(0.0008)				
**Motility/adhesion**											
**L**	**EFNA3**	**1.49**	*0.0249*								
			(0.1881)								
R	EPHA4	**2.22**	*0.0032*	**2.00**	*0.0001*	**3.33**	*0.0002*			**1.43**	*0.0217*
			(0.0680)		(0.0079)		(0.0008)				(0.2159)
**L**	**EFNA4**			**1.67**	*0.0166*						
					(0.1094)						
R	EPHA4	**2.22**	*0.0032*	**2.00**	*0.0001*	**3.33**	*0.0002*			**1.43**	*0.0217*
			(0.0680)		(0.0079)		(0.0008)				(0.2159)
**L**	**LGALS1**			**2.00**	*0.0140*	**2.50**	*0.0000*	**2.00**	*0.0002*	**2.00**	*0.0026*
					(0.0984)		(0.0001)		(0.0036)		(0.0769)
R	SPN			**1.25**	*0.0147*						
					(0.1016)						
**L**	**PLAU**	**4.35**	*0.0005*	**2.50**	*0.0232*	**2.40**	*0.0001*	**2.50**	*0.0003*	**3.33**	*0.0006*
			(0.0236)		(0.1322)		*(0.0001)*		(0.0043)		(0.0486)
R	PLAUR			**1.25**	*0.0069*	**3.33**	*0.0000*				
					(0.0638)		(0.0001)				
**L**	**SEMA3F**	**2.33**	*0.0216*	**1.25**	*0.0183*	**1.67**	*0.0055*			**1.25**	*0.0496*
			(0.1783)		(0.1159)		(0.0134)				(0.3115)
R	NRP2	**4.76**	*0.0028*	**2.50**	*0.0038*	**2.50**	*0.0000*			**1.11**	*0.0390*
			(0.0651)		(0.0478)		(0.0001)				(0.2704)
**L**	**SEMA3B**			**1.11**	*0.0307*						
					(0.1575)						
R	NRP2	**4.76**	*0.0028*	**2.50**	*0.0038*	**2.50**	*0.0000*			**1.11**	*0.0390*
			(0.0651)		(0.0478)		(0.0001)				(0.2704)
**L**	**SPP1**			**2.50**	*0.0015*	**3.33**	*0.0001*	**2.00**	*0.0003*	**2.00**	*0.0024*
					(0.0271)		(0.0005)		(0.0043)		(0.0769)
R	ITGAV			**1.25**	*0.0441*			**1.67**	*0.0008*	**1.43**	*0.0059*
					(0.1955)				(0.0050)		(0.1150)
R	CD44	**2.22**	*0.0043*	**1.67**	*0.0022*	**2.00**	*0.0000*	**1.67**	*0.0064*	**1.67**	*0.0009*
			(0.0799)		(0.0352)		(0.0000)		(0.0251)		(0.0486)
R	ITGA9	**1.59**	*0.0282*			**1.67**	*0.0032*				
			(0.2010)				(0.0087)				
											
R	ITGB1					**1.67**	*0.0022*				
							(0.0063)				

1 Modulation was calculated as mean ratio of paired tumor (T)/controlateral normal tissue (N) gene expression. An arbitrary threshold of **Ligand** or Receptor gene expression modulation was settled at a *P*<0.01 (paired t-test) and/or FDR <0.25 and the results were considered relevant if present in at least 3 datasets. Above this arbitrary threshold, all the possible pairings with this L or R are reported. Significant ratios are in bold. The gaps mean that the T/N ratios were below the arbitrary significant threshold.

Among the downregulated L/R pairs were genes involved in angiogenesis (ANGPTL1/TEK, 2 datasets; VEGF/FLT1, 2 datasets) and growth pathways (BMP2/BMPR1A, 2 data sets; FGF13/FGFR2, 2 datasets).

Interestingly, the ligands PLAU and SPP1 have previously been associated with invasion and progression in several types of solid tumors, and both in vivo and in vitro models have demonstrated overexpression of these ligands in thyroid tumors [12], [13].

**Figure 1:**
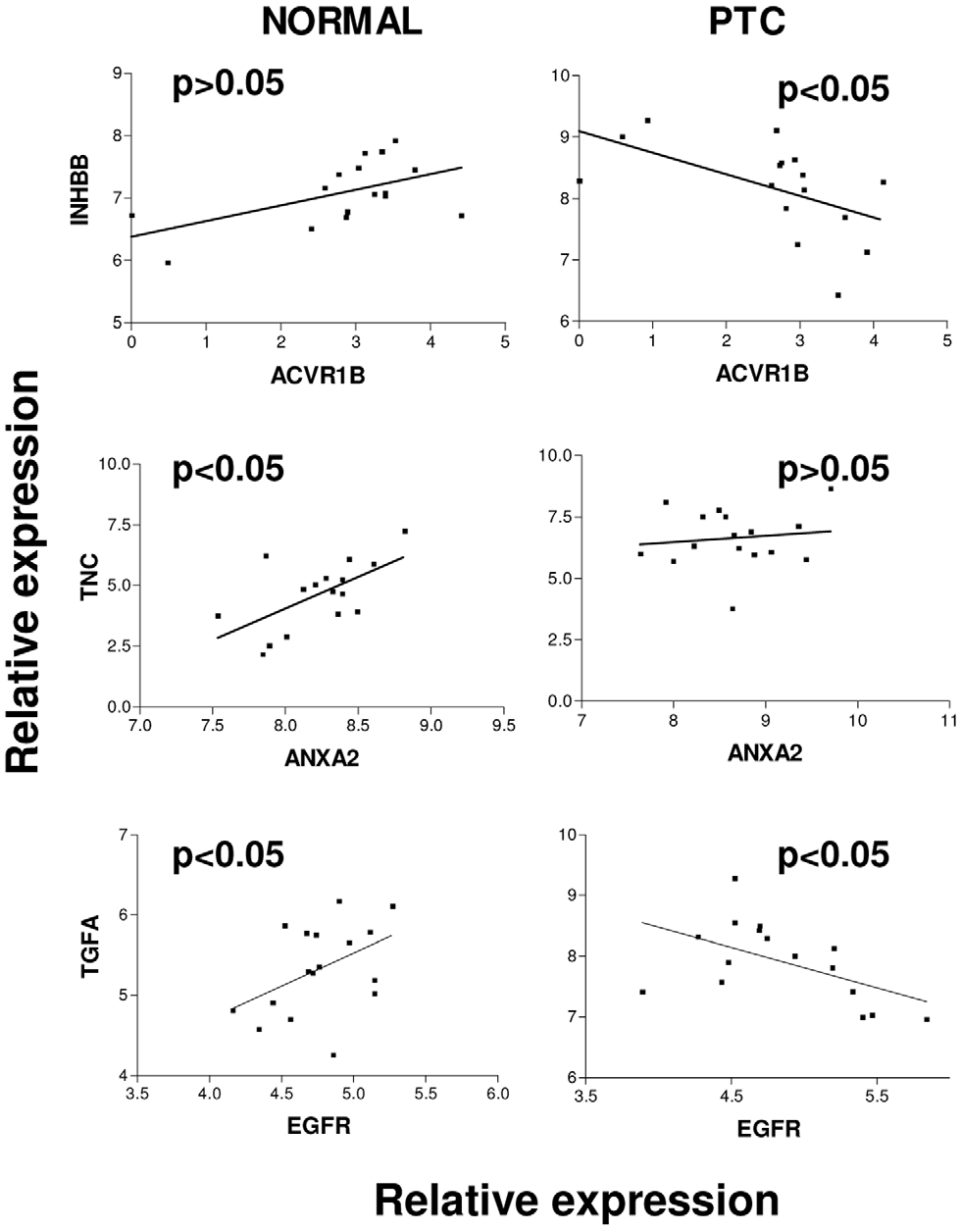
Analysis of L/R pairs differentially expressed in normal or tumor thyroid samples. Correlation analysis of INHBB/ACVR1, TNC/ANXA2, and TGFA/EGFR pairs in normal contralateral or thyroid cancer samples of dataset III (see Table 1) obtained by Pearson correlation. *P* values are indicated.

To test how the expression of the three L/R pairs involved in growth pathways, INHBB/ACVR1, TNC/ANXA2, and TGFA/EGFR, was regulated among 16 normal thyroid or 23 PTC samples, a correlation analysis was performed between the pairs in dataset III, which included the largest number of samples (see Table 1). A significant inverse correlation was observed in the thyroid cancer samples for the L/R pair INHBB/ACVR1 (P<0.05) (Fig. 1), but not in the normal samples. The expression of the TNC/ANXA2 pair was directly correlated (P<0.05) only in normal thyroid samples. For TGFA/EGFR, a significant direct and inverse correlation was observed in normal and tumor samples, respectively.

All together, these data show that the expression of INHBB, ANXA2, and TGFA ligands could be specifically up-modulated in PTCs vs. normal contralateral cells, thus supporting the hypothesis that their differential expression is finely regulated in normal tissues vs. tumor tissues. The signaling loops associated with the pairs INHBB/ACVR1, TNC/ANXA2, and TGFA/EGFR appeared to be relevant in the pathogenesis of PTCs.

### EGFR-associated signaling loops are concordantly up-modulated in RET/PTC1-infected thyrocytes and tumor samples

Based on previous evidence from our laboratory that autocrine signaling loops are detectable in RET/PTC1-infected thyrocytes [6], we decided to test the relevance of the autocrine loops by comparing the L/R pairs differentially expressed in PTCs vs. normal contralateral tissues (see Table 2) with those differentially expressed in RET/PTC1-infected thyrocytes vs. normal thyrocytes [6]. We identified 10 L/R pairs that showed concordant expression between the two analyses (Table 3). Among these, four EGFR ligand/EGFR pairs emerged, and in particular, TGFA was robustly upregulated in the in vitro model (14.22-fold) as well as in the PTC tumor samples (range: 3.3–5 fold). Elevated AREG expression (148,50-fold increase) was observed in RET/PTC1-infected thyrocytes with respect to normal thyrocytes. PLAU/PLAUR, SPP1/CD44, and SPP1/ITGA9 were concordantly up-regulated in the RET/PTC1-infected vs. normal thyrocytes accordingly to that observed for PTC samples compared with their normal counterparts.

**Table 3:** Comparison between the in vitro model and the PTCs/normal samples.

L/R	Gene Symbol	RET_PTC1/Normal	T/N*				
			I	II	III	IV	V
***Concordant***							
**L**	**ANGPTL1**	0.09	0.20	0.21			
R	TEK	0.19	0.70	0.63	0.63		
**L**	**AREG**	148.50			2.00		
R	EGFR	2.42		1.67	1.67	1.25	
**L**	**BTC**	9.46		1.25			
R	EGFR	2.42		1.67	1.67	1.25	
**L**	**DTR**	4.47			2.50		
R	EGFR	2.42		1.67	1.67	1.25	
**L**	**TGFA**	14.22	4.17	3.33	5.00		
R	EGFR	2.42		1.67	1.67	1.25	
**L**	**FGF7**	0.13			0.63		
R	FGFR2	0.03	0.40	0.43	0.26	0.83	
**L**	**EFNA4**	36.76		1.67			
R	EPHA4	2.59	2.22	2.00	3.33		1.43
**L**	**PLAU**	21.22	4.35	2.50	10.00	2.50	3.33
R	PLAUR	4.63		1.25	3.33		
**L**	**SPP1**	3.52		2.50	3.33	2.00	2.00
R	CD44	3.70	2.22	1.67	2.00	1.67	1.67
**L**	**SPP1**	3.52		2.50	3.33	2.00	2.00
R	ITGA9	4.64	1.59		1.67		
***Discordant***							
**L**	**CXCL12**	2.59	0.20	0.45	0.29	0.42	
R	CXCR4	107.10	0.40				
**L**	**TNFSF10**	3.55	0.40		0.50	0.77	
R	TNFRSF11B	2.23	0.10	0.19	0.18		
**L**	**PGF**	4.34	0.30		0.40		
R	NRP1	5.61		0.77			
**L**	**TGFB2**	0.28		1.25			
R	TGFBR1	0.48		1.43			

*T/N values are from Table 2. See Table 2 legend for selection criteria. Concordant or discordant L/R pairs between the in vitro model and clinical samples.

Within the discordant L/R pairs, CXCL12/CXCR4 pair emerged upregulated in RET/PTC1-infected thyrocytes with respect to normal thyrocytes [6] and down-modulated in PTC samples vs. the normal counterpart.

In summary, bioinformatic analysis identified frequent upregulation of the L/R pair TGFA/EGFR in both RET/PTC1-infected thyrocytes as well as in PTC samples with respect to their normal counterparts, supporting the notion of an autocrine TGFA/EGFR loop of signaling in PTCs.

### Validation of TGFA and EGFR expressions in PTCs and RET/PTC1-infected thyrocytes

**Figure 2:**
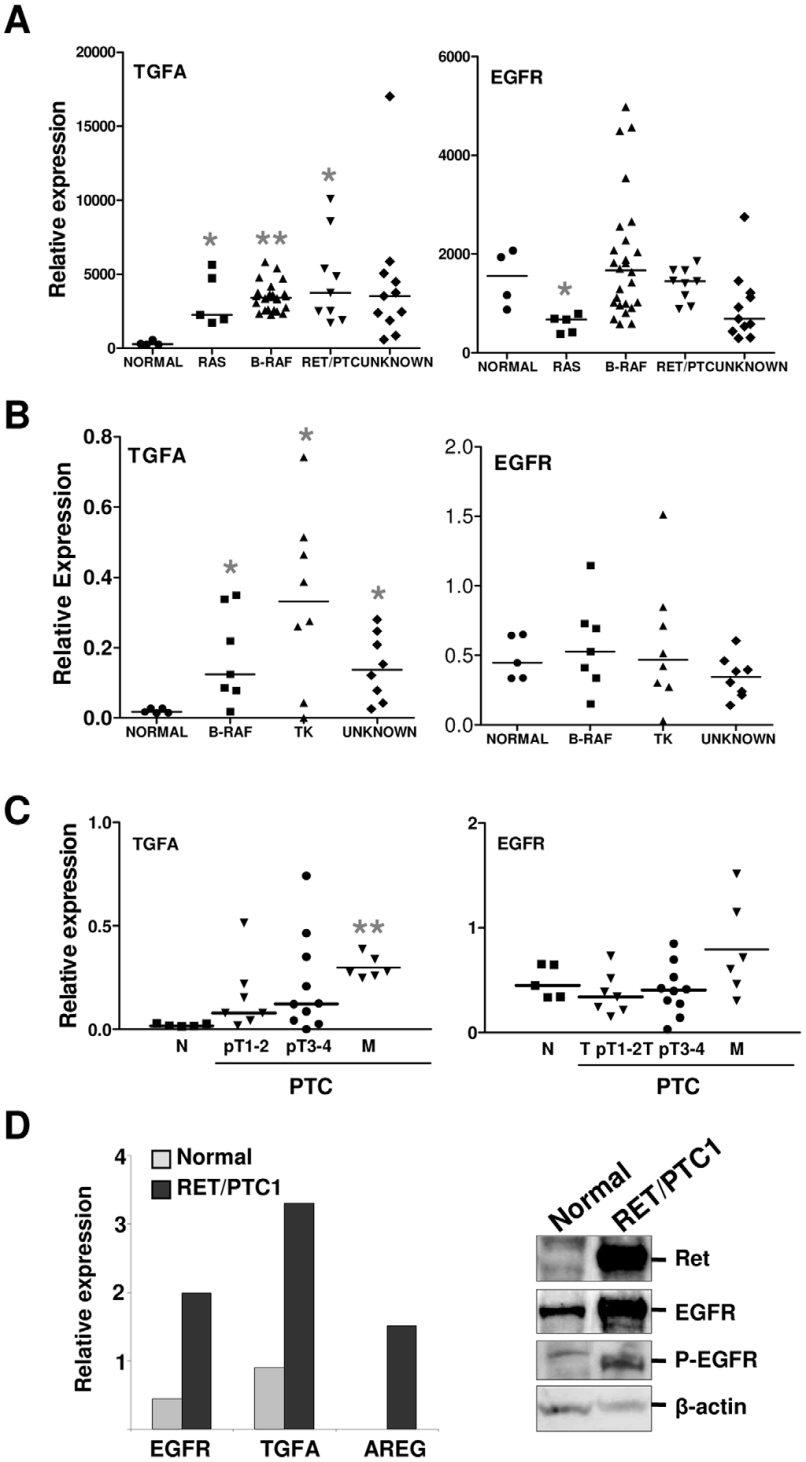
Validation of TGFA and EGFR expressions in PTCs. **A**. Analysis of TGFA and EGFR expression levels in dataset presented by Giordano et al. [9]. Results are shown as relative expression. *, P<0.05 and **, P<0.0001: statistically significant results in comparison with normal samples (P values were determined by two-tailed Student's t-test with unequal variance). **B**. Real-time RT-PCR analysis of TGFA and EGFR genes in normal thyroid (from pathologies other than cancer) and PTC specimens. Results are given as relative expression (mRNA expression normalized for PGK1 mRNA levels). Medians are shown. *, P<0.05: statistically significant results in comparison with normal samples (P values were determined by two-tailed Student's t-test with unequal variance). **C**. Real-time RT-PCR analysis of TGFA gene in the same specimens reported in panel B, classified according to tumor staging. pT1, tumors less than 1 cm and limited to the thyroid; pT2, tumors more than 1 cm but not more than 4 cm in greatest dimension and limited to the thyroid gland; pT3, tumors more than 4 cm and limited to the thyroid; pT4, tumors displaying local extrathyroid spread; and M, lymph nodal metastasis. **, P<0.0001: statistically significant result in comparison with normal samples (P values were determined by two-tailed Student's t-test with unequal variance). **D**. Left panel: Real-time RT-PCR analysis of EGFR, TGFA, and AREG genes in normal thyreocytes and RET/PTC1-infected cells. Results are presented as relative expression (mRNA expression normalized for PGK1 mRNA levels). Right panel: biochemical analysis of parental and RET/PTC1-infected thyrocytes. Cell extracts were analyzed with the following Abs: anti-RET (ret), anti-EGFR (EGFR), anti phosphorylated EGFR (P-EGFR). β-actin is shown as a control for protein loading. One representative experiment of three is shown.

We next analyzed TGFA and EGFR expression levels using a dataset of PTC samples with known genetic mutations [9]. It is worth noting that in this study, the normal thyroid tissues were from pathologies other than thyroid cancer. TGFA expression was significantly up-modulated in RAS-, BRAF-, and RET/PTC1-mutated PTCs with respect to normal thyroid tissues (Fig. 2A, left panel). No differences were observed for EGFR, with the only exception of RAS-mutated PTCs (Fig. 2A, right panel).

Subsequently, real-time RT-PCR analysis of TGFA and EGFR transcripts was performed on total RNA from 5 normal thyroid tissue samples from pathologies other than thyroid cancer and 23 PTC biopsies (Table S1). TGFA transcripts were significantly increased in all PTCs relative to normal thyroid samples (Fig. 2B, left panel). No significant differences in EGFR expression were detectable between the normal and tumor samples, as well as within all PTCs tested with different genetic lesions (Fig. 2B, right panel). The TGFA and EGFR expressions of the same samples were then compared subdividing the tumors in primary lesions (stage 1–2 and stage 3–4) and lymph nodal metastases (Fig. 2C). TGFA transcripts were significantly higher in PTC metastases when compared with normal thyroid samples, and although not significant, TGFA levels trended upwards with tumor stage (Fig. 2C, left panel). In addition, EGFR transcripts were also trended upwards, although not significant (Fig. 2C, right panel).

We next performed real-time RT-PCR for TGFA and EGFR on total RNA from normal and RET/PTC1-infected thyrocytes. In agreement with the gene expression analysis (see Table 3), TGFA and EGFR transcripts were increased to about 3.5- and 5-fold in RET/PTC1-infected thyrocytes relative to normal cells (Fig. 2D, left panel). Consistent with the bioinformatic analysis, AREG transcript was de novo expressed at detectable levels in RET/PTC1-infected thyrocytes. In addition, Western blot analysis identified elevated EGFR protein and phosphoprotein in total cell lysates from RET/PTC1-infected thyrocytes in comparison with the lysates from normal thyrocytes (Fig. 2D, right panel).

These data essentially validated the bioinformatic analysis demonstrating the up-modulation of TGFA in PTCs, in RET/PTC1-infected thyrocytes, and in the advanced stages of PTC progression.

### Majority of PTC cell lines expressed high levels of both TGFA and EGFR

**Figure 3:**
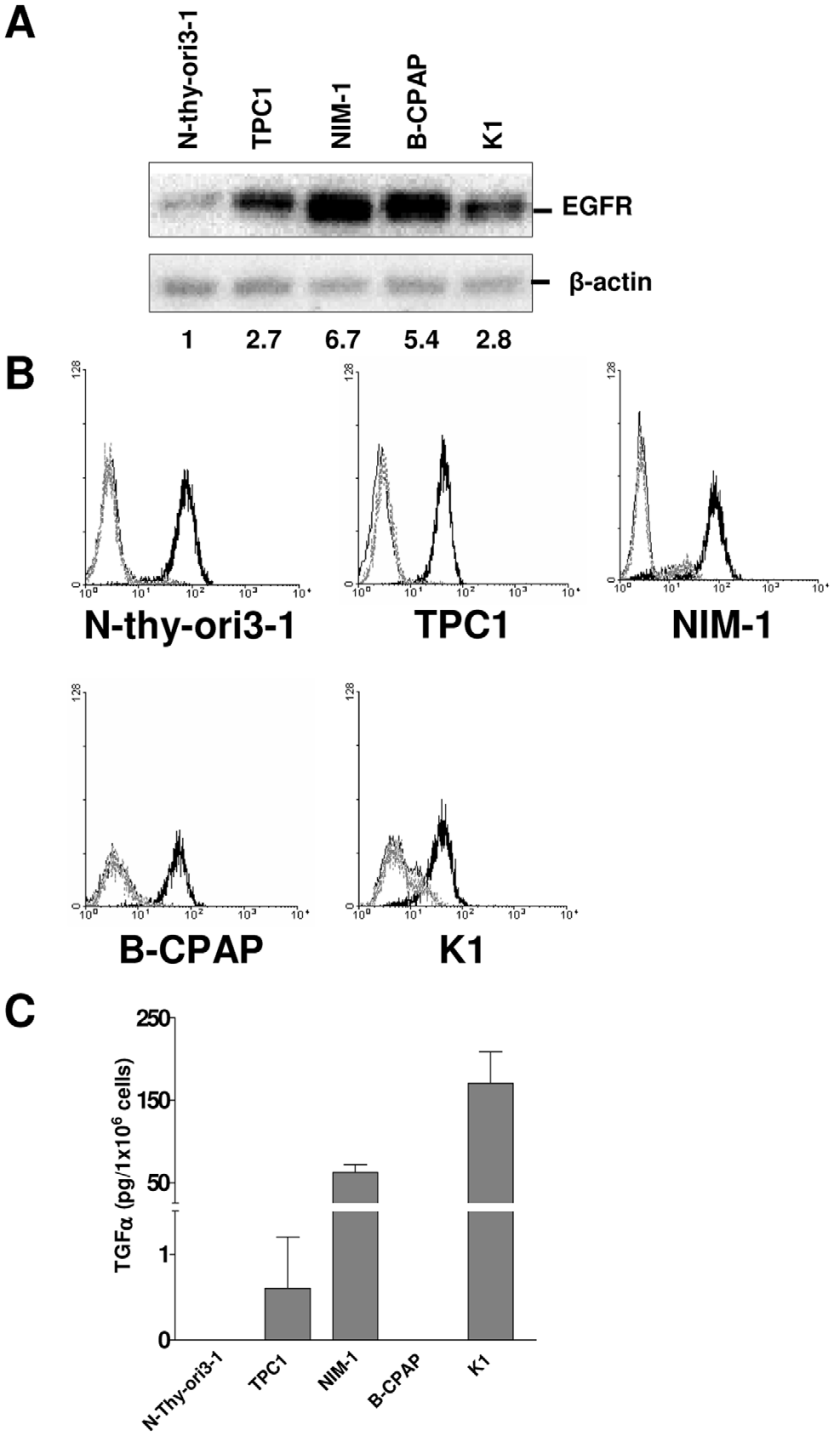
Majority of PTC cell lines expressed high levels of both EGFR and TGFA. **A.** Western blot analysis with anti-EGFR Ab was performed on total cell lysates from PTC cell lines. Values corresponding to the densitometric evaluation of EGFR expression are shown at the bottom. Abs used are indicated. β-actin is shown as a control for protein loading. **B.** FACS analysis on PTC cell lines stained with anti-EGFR Ab (dark black peak), with a mouse IgG1 isotype control (grey dotted peak) or only with the secondary labelled Ab (black peak). **C.** TGFA production by thyroid cell lines. Media without FBS were conditioned for 48 h by confluent cells and analyzed by ELISA. One representative experiment of two is shown.

To elucidate the functional role of TGFA/EGFR, we analyzed their protein levels in four PTC cell lines with known and determined genetic lesions (see Table S2). An immortalized thyrocyte cell line, N-Thy-ori3-1 [14], and the HeLa cell line were included as controls for EGFR-expressing nontransformed and tumor cells, respectively. Western blot analysis on total cell lysates with anti-EGFR antibody (Ab) demonstrated EGFR expression in all the cell lines tested, with TPC1, NIM-1, and B-CPAP expressing the highest levels (Fig. 3A). FACS analysis with anti-EGFR Ab showed comparable membrane expression of EGFR on N-Thy-ori3-1 and in PTC cell lines (Fig. 3B). The apparent discrepancy between the EGFR protein levels in western blot and FACS analyses might be due to EGFR internalization and accumulation in the cytoplasm in PTC cells.

Subsequently, we used ELISA to detect TGFA in conditioned medium from the above-tested cells (Fig. 3C). NIM-1 and K1 conditioned media contained 50 and 150 pg/10^6^ cells of TGFA, respectively, and TPC1 medium contained low but evaluable amount of TGFA. The conditioned media from the immortalized N-Thy-ori3-1 thyrocyte and B-CPAP cell lines contained undetectable levels of TGFA. As TGFA could not be completely released from the cell membrane [15], we performed ELISA also on total lysates from the same cell lines, but only trace amount of endogenous TGFA was detectable in all the cell lines (data not shown). On the other hand, B-CPAP cells appeared to express AREG ligand by real-time RT-PCR (data not shown).

Thus, in vitro analysis showed that both immortalized thyrocytes and PTC cell lines express EGFR but only PTC cells secrete TGFA.

### EGFR and TGFA are functional in the NIM-1 thyroid cancer cell line

**Figure 4:**
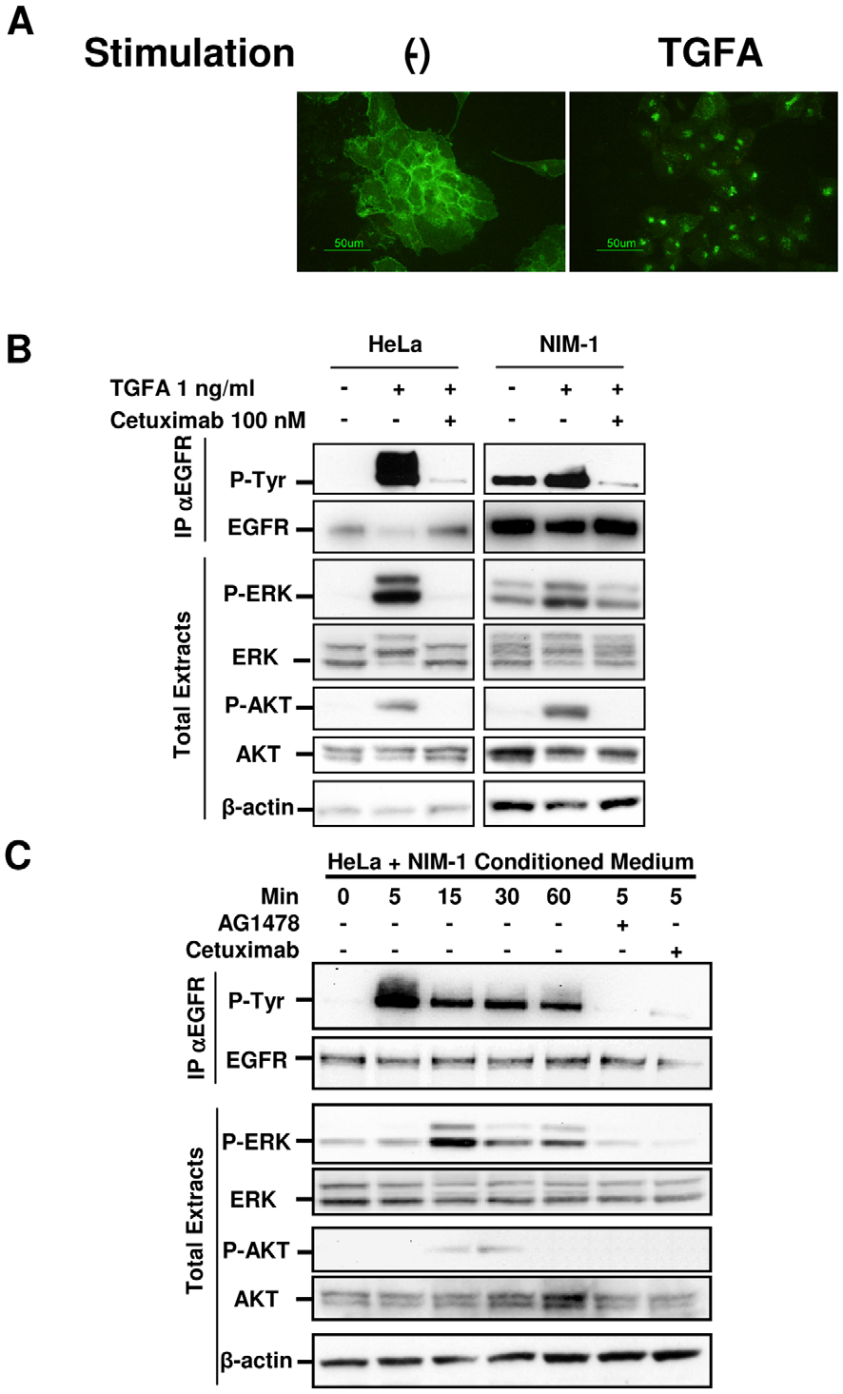
EGFR and TGFA are functional in NIM-1 thyroid cancer cell line. **A.** IF analysis with anti-EGFR Ab on serum-starved NIM-1 cells left untreated or treated with 100 ng/ml TGFA for 24 h. **B.** Biochemical analysis on HeLa and NIM-1 cell extracts. After serum starvation for 24 h, the cells were left untreated or treated with 100 nM Cetuximab, or control chimeric Ab (data not shown) for 2 h, and then stimulated with 1 ng/ml TGFA for 5 min. Abs used are indicated. EGFR phosphorylation has been analyzed by immunoprecipitation (IP) with anti-EGFR Ab and western blotting with anti-phosphotyrosine Ab (P-Tyr). β-actin is shown as a control for protein loading. **C.** Western blot analysis on HeLa cell extracts. After serum starvation for 24 h, the cells were exposed to fresh medium without FBS (-) or to conditioned medium of NIM-1 cells for 1 h. Pretreatment with AG1478, Cetuximab, or control chimeric Ab (data not shown) was performed for 2 h, and then NIM-1 conditioned medium was added to the culture. As control of specific Ab inhibition, the cells were treated with an unrelated human Ab. Abs used are indicated. β-actin is shown as a control for protein loading. One representative experiment of three is shown.

To evaluate the ability of the TGFA/EGFR signaling loop to transduce signals in PTCs, NIM-1 cells were chosen as an in vitro model. It is known that when TGFA interacts with EGFR, the complex is internalized and eventually degraded in lysosomes [16]. Indeed, immunofluorescence (IF) analysis of fixed NIM-1 cells using anti-EGFR Ab showed that in starved cells, EGFR was mostly localized on the cell membrane (Fig. 4A, left panel). After 24 h of TGFA stimulation, EGFR expression was detectable only in small intracellular punctuate vesicles (right panel), suggesting an endocytic mechanism of receptor degradation. These results suggest that ligand-induced EGFR activation elicited the internalization and intracellular sorting of the TGFA/EGFR complex.

Subsequently, we treated starved cells with 1 ng/ml of TGFA in the absence or presence of Cetuximab, a chimeric anti-EGFR Ab known to inhibit ligand binding to EGFR with 10-fold higher affinity than endogenous ligands (either EGF or TGFA) [17], and analyzed EGFR phosphorylation by immunoprecipitation (IP) with anti-EGFR Ab and Western blotting with anti-phosphotyrosine (P-Tyr) Ab. EGFR was already phosphorylated in 24-h-starved NIM-1 cells, but following TGFA stimulation, its phosphorylation level was increased (Fig. 4B). Cetuximab pretreatment lowered EGFR phosphorylation to levels below those observed in starved cells. In HeLa cells, EGFR phosphoprotein was detectable only after TGFA treatment. The basal EGFR phosphorylation observed in NIM-1 cells is consistent with the hypothesis that endogenous TGFA contributes to EGFR phosphorylation. Furthermore, EGFR phosphorylation was associated with ERK and AKT phosphorylation, and Cetuximab pretreatment reduced this phosphorylation to levels similar to those observed in starved cells, indicating that the MEK/ERK and PI3K/AKT pathways depend at least in part on EGFR activation (Fig. 4B).

Starved HeLa cells did not produce detectable amount of TGFA (data not shown) and showed no EGFR basal phosphorylation. We subsequently tested the capability of NIM-1-conditioned medium to activate EGFR in these cells. EGFR phosphorylation was detectable after 5-min stimulation, decreased slightly after 15-min stimulation, and remained stable after 60-min treatment. ERK phosphorylation reached a maximum after 15 min of NIM-1-conditioned medium treatment and maintained these levels for up to 60-min treatment (Fig. 4C). Phospho-AKT increased slightly after 15-min treatment, reaching a maximum after 30 min before disappearing after 60-min treatment. Pretreatment with either the tyrosine kinase inhibitor AG1478 or Cetuximab blocked stimulation of EGFR phosphorylation. These data support the presence of functional EGFR ligand/s, possibly TGFA (see Fig. 3C), in the NIM-1-conditioned medium. Altogether, these results show in vitro that TGFA/EGFR signaling loop activates both MEK/ERK and PI3K/AKT pathways in PTC cells.

### TGFA/EGFR signaling contributes to the proliferation of PTC cells

**Figure 5:**
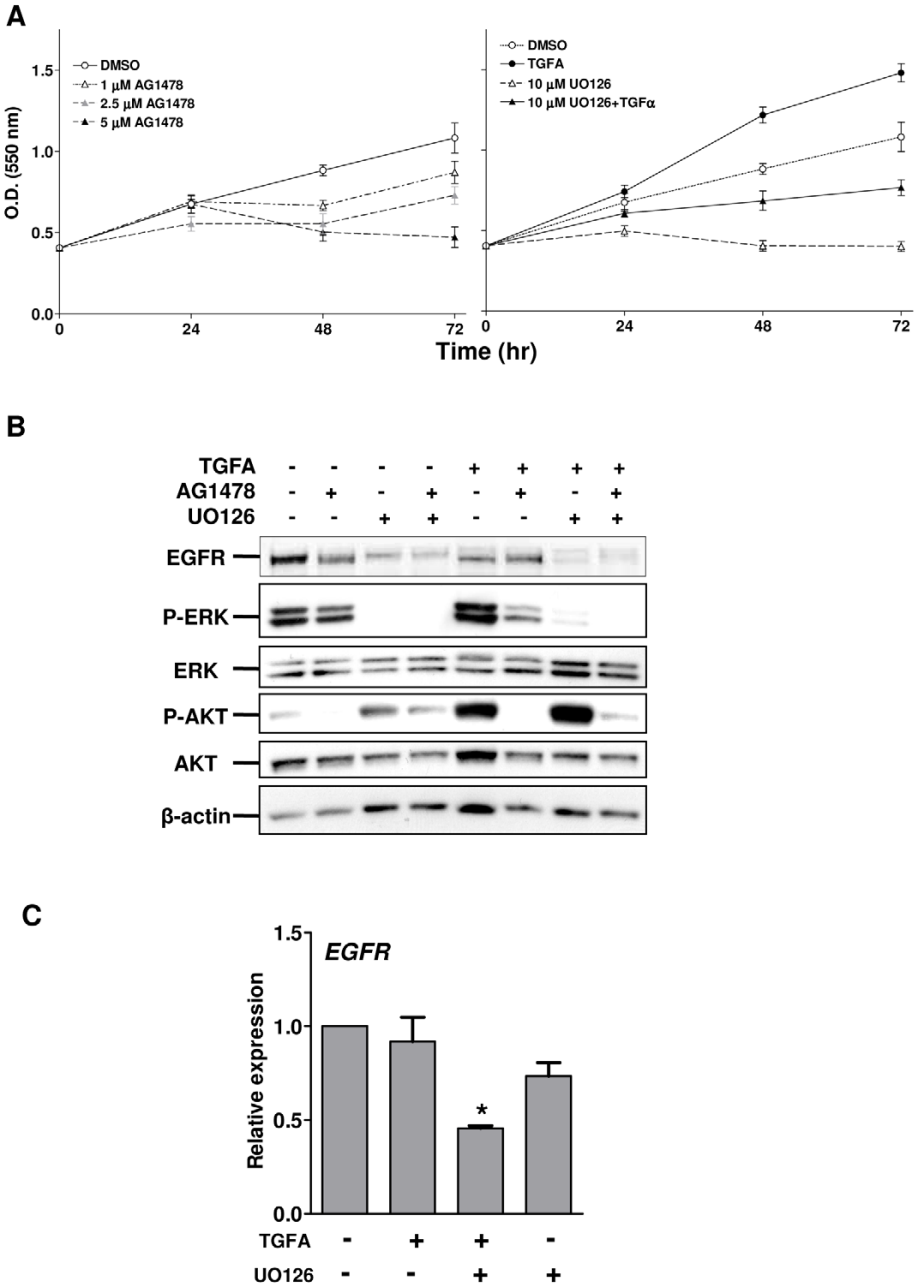
TGFA/EGFR signaling contributes to the proliferation of PTC cells. **A.** NIM-1 cells were exposed to solvent or to different drugs for up to 72 h in medium without FBS. Cell proliferation was evaluated by SRB assay. Representative growth curves are shown. Each point represents the mean of eight independent replicates ± SD. **B.** Western blot analysis was performed on total cell lysates from starved NIM-1 cells unstimulated or stimulated with 100 ng/ml of TGFA for 24 h. When indicated, the cells were also treated with AG1478 and/or UO126. Abs used are indicated. β-actin is shown as a control for protein loading. One representative experiment of four is shown. **C.** Real-time RT-PCR analysis of EGFR gene in NIM-1 cells treated with the MEK1/2 inhibitor UO126. Serum-starved cells were exposed to solvent (-) or to 10 µM UO126 in the presence of 100 ng/ml of TGFA. Total RNA was extracted after 24 h. The results are presented as relative expression (mRNA expression normalized for PGK1 mRNA levels). *, P<0.05: statistically significant result in comparison with cells exposed to solvent (*P* value was determined by two-tailed Student's *t-*test with unequal variance).

We next analyzed whether activated EGFR could be involved in cell proliferation by monitoring NIM-1 cell growth for up to 72 h in the absence or presence of AG1478. AG1478 decreased cell proliferation in a dose-dependent manner, with the highest AG1478 dose apparently toxic after 48-h treatment (Fig. 5A, left panel). As the NIM-1 cell line harbors a BRAF V600E mutation, with the result that the MEK/ERK pathway is constitutively activated, we investigated whether TGFA/EGFR signaling in this cell line was dependent on RAF/MEK/ERK activation by inhibiting MEK-dependent proliferation using the MEK inhibitor UO126. An increase in the growth rate of starved NIM-1 cells was detectable after 24-, 48-, and 72-h treatment with TGFA (Fig. 5A, right panel). UO126 totally blocked cell growth, and TGFA stimulation rescued growth capability, although the growth rate did not reach a level similar to that of TGFA-stimulated cells (Fig. 5A, right panel). Therefore, EGFR stimulation by TGFA might be able to sustain PTC cell proliferation independent of the MEK/ERK pathway.

To define the signaling pathway activated by TGFA/EGFR loop when the MEK/ERK pathway was blocked, Western blot analysis was performed on total cell lysates from 24-h-starved NIM-1 cells stimulated for 24 h with TGFA alone or together with UO126. In this experiment, AG1478 treatment was also included to inhibit the tyrosine kinase activity of EGFR. In starved NIM-1 cells, phospho-ERK decreased upon UO126 treatment, while phospho-AKT slightly increased (Fig. 5B). Phospho-ERK decreased only slightly in AG1478-treated cells. Furthermore, 24-h TGFA stimulation induced both phospho-ERK and -AKT, an effect offset by AG1478 treatment (Fig. 5B). Interestingly, stimulation with TGFA in the presence of UO126 completely abolished phospho-ERK, while phospho-AKT strongly increased; treatment with both AG1478 and UO126 reversed this effect, indicating that AKT phosphorylation is at least partially dependent on EGFR phosphorylation. Total EGFR expression decreased when the cells were treated with UO126, suggesting that RAF/MEK/ERK pathway, constitutively activated in this cell line, might regulate EGFR expression at transcriptional and/or protein levels. Treatment with the PI3K inhibitor Ly294002 completely abolished phospho-AKT, indicating its dependency on PI3K activation (data not shown). PI3K/AKT activation was not owing to Her-3 expression, because Her-3 was never expressed in NIM-1 cells (data not shown).

To investigate whether MEK inhibition could affect EGFR expression at transcriptional levels, real-time RT-PCR was performed on total RNA from NIM-1 cells treated with UO126 as shown earlier. EGFR expression decreased up to 2-fold in cells treated with TGFA and UO126 (Fig. 5C). The results support the hypothesis that constitutively activated RAF/MEK/ERK pathway modulates not only protein stability but also EGFR transcript.

Altogether, these data demonstrate that TGFA/EGFR signaling loop contributes to the growth of PTC cells, possibly by sustaining PI3K/AKT pathway activation.

## Discussion

In this study, we applied a combined bioinformatic and biological approach to the analysis of signaling in PTC and found that: i) the TGFA/EGFR pair emerged as one of the most tightly regulated L/R pair as assessed by gene expression analysis in a total of 92 PTC samples (41 of Table 1 and 51 from the dataset obtained from the study by Giordano *et al*. [9]) and 41 contralateral tissues; ii) tumor thyroid tissues, as assessed by real-time RT-PCR, expressed similar levels of EGFR transcripts respect to normal thyroid tissues, but the levels of TGFA transcripts were significantly elevated in PTCs, in particular those with BRAF and RET/PTC mutations, and in larger or metastatic tumors; iii) RET/PTC1 expression was associated with increased EGFR and TGFA expressions in RET/PTC1-infected thyrocytes; and iv) TGFA/EGFR signaling increased the growth of a BRAF-mutated PTC cell line, possibly through PI3K/AKT activation, independently from MEK/ERK signaling.

We found potentially activated L/R signaling loops in PTC by interrogating a previously used L/R database [11]. As a proof-of-principle, we had already applied the same bioinformatic approach to ovarian cancer, but a list of the L/R pairs coordinately expressed in tumors vs. normal ovary tissues was not compiled, owing to the impossibility to have gene expression data of normal tissue from the same ovarian cancer patient. The major advantage in applying this bioinformatic tool to PTC resides in the ability to compare coordinated L/R expressions in PTC vs. the normal counterpart, thus highlighting only those L/R pairs truly specific for tumor cells from thyroid origin in an intra-patient analysis. Some internal validations emerged, such as the pairs PLAU/PLAUR, SPP1/CD44, and SPP1/ITGA9, whose ligands have been demonstrated overexpressed in thyroid tumors [12], [13] as well as in our in vitro model [6]. Furthermore, other L/R pairs identified in our analysis, namely INHBB/ACVR1 and TNC/ANXA2, have been shown to be involved in invasiveness of other solid tumors [18], [19], and as they have not yet been studied in thyroid cancer, warrant further investigation in this context. The correlation analysis among PTCs (see Fig. 1) highlighted an inverse correlation between INHBB/ACVR1 and TGFA/EGFR pairs. Note that the analysis reported in Table 2 refers to the up- or down- modulation in PTCs respect to the normal controlateral thyroid tissues within the same patient while the analysis shown in Fig. 1 reports the correlation between the ligand and its receptor separately in normal thyroid tissues or in PTC samples. These data suggest that within the tumor the increased ligand expression could down-modulate the expression of its receptor. The latter observation is not in contrast with the overall slightly increased receptor expression when comparing tumors to their normal counterpart. Previous analysis of EGFR expression in thyroid cancer also gave controversial results: some studies report up-regulation in thyroid carcinomas, particularly in ATC, whereas others report expression levels similar to those observed in normal tissue (reviewed in [20]).

Within the biological classes of L/R pairs identified by our analysis, the most represented class was that associated with growth. Few association were observed with chemo/cytokine-genes in PTCs, and in particular, the CXCL12/CXCR4 pair was found to be downregulated in PTCs vs. normal contralateral tissues. We had previously shown that de novo RET/PTC1 expression in normal thyrocytes induces a pro-inflammatory program [6], and Castellone *et al*. showed that CXCR4 is expressed in PTCs but not in normal thyroid tissues [21]. The discrepancy between these studies and the current one could be explained by the fact that while the expression of an oncogene, such as RET/PTC1, is sufficient per se to induce the CXCL12/CXCR4 pair and other inflammation-related genes *in vitro*, a combination of mechanisms may be required to regulate these genes in tumors. Moreover, our analysis compared gene expression of PTCs with respect to the normal contralateral counterpart, which could have a transcript profile different from that of the normal thyroid tissue.

From the several L/R pairs that emerged from our analysis, we chose the TGFA/EGFR signaling loop for further validation. TGFA and EGFR have been previously found to be co-expressed in thyroid cancer and have been associated with a more aggressive disease [22], [23]. Recently, gene expression analyses have identified TGFA upregulation in thyroid carcinomas, especially in PTCs [24], [25]. In particular, Delys *et al.* showed a statistically significant alteration of the EGF signaling pathway as well as other genes involved in this cascade [26]. However, prior to the current study, a functional TGFA/EGFR signaling loop has not been experimentally demonstrated in thyroid cancer. Here, we have also showed that EGFR expression is not up-regulated in PTC samples respect to normal thyroid tissues from pathologies other than thyroid cancer (see Fig. 2). This discrepancy might be due to a different and fine regulation of EGFR expression in PTC cells respect to normal controlateral thyroid cells. Interestingly, we observed increasing TGFA expression levels in high-stage PTCs as well in metastasis. These results, together with the fact that NIM-1 cells derived from a PTC metastasis to the sacral bone, await further analysis on a large number of PTC samples to validate a possible association between TGFA expression and PTC progression. On the other hand, PTC samples and cell lines expressing low TGFA levels might represent a PTC subtype expressing other EGFR ligands such as AREG, as already observed [26].

Our data provide several lines of evidence that a TGFA/EGFR signaling loop is significantly associated with PTC harboring BRAF and RET/PTC mutations. So far, it is known that both alterations lead to the activation of common downstream MAP kinase pathway [5]. In this study, we showed that in NIM-1 cells the MEK kinase inhibitor UO126 impaired cell growth. Interestingly, in TGFA-stimulated NIM-1 cells, PI3K/AKT signaling increased after the inhibition of MEK pathway, and was decreased by the tyrosine kinase inhibitor AG1478 together with the MEK inhibitor UO126. These results argue that PI3K/AKT pathway is partially dependent on TGFA/EGFR signaling, an observation that could have important implications in developing therapeutic PTC compounds directed against targets other than the MEK/ERK pathway. Furthermore, it seems envisagable that the same mechanism could also be active in other tumors with high BRAF mutation frequency, such as melanomas [27].

Recently, RET/PTC was found to be associated with EGFR in thyroid PCCL3 cells, thus contributing to cell growth by modulating RET/PTC phosphorylation and conditional RET/PTC expression up-modulated EGFR protein and phosphoprotein levels [28]. Consistent with these findings, we have shown here that in RET/PTC1-infected thyrocytes, EGFR expression was upregulated at the transcriptional level. We also observed that EGFR transcript levels were significantly lower in RAS-mutated PTC when compared with the normal counterpart. These results are consistent with the fact that RAS mutations are mostly present in the follicular variant of PTCs and that this mutation correlates with specific gene expression profiles [9]. Indeed PTCs harboring RAS mutation show significantly less prominent nuclear features of the tumor, more frequent encapsulation, and low-rate lymph node metastases [4].

Interference with EGFR activation has been previously considered in the therapy of thyroid cancers. A phase II study of gefitinib in patients with advanced thyroid cancer showed that the drug was poorly effective in curing patients – disease stabilization was observed in only 32% of patients [29]. In this study, we have shown that constitutive active MEK/ERK signaling, owing to BRAF mutation, may function in tandem with EGFR signaling. Thus, EGFR intervention alone seems to be insufficient in the treatment of thyroid cancer.

In conclusion, compiling a reliable list of L/R pairs associated with PTC progression and validating the biological role of the autocrine TGFA/EGFR loop in thyroid cancer, in vitro, have enabled us to better understand the biology of BRAF- and RET/PTC-mutated PTCs and to identify potential new tools for diagnosis and therapeutic intervention.

## Materials and Methods

### Ethics statement

All patients whose biological samples were included in the study signed an informed consent, approved by the Independent Ethical Committee of the Fondazione IRCCS Istituto Nazionale dei Tumori, Milano (Italy), to donate the leftover tissue specimens after completing diagnostic procedures to the Fondazione IRCCS Istituto Nazionale dei Tumori for research purposes.

### Bioinformatic analysis

We interrogated 5 datasets of gene expression profiles from thyroid carcinoma samples with a list of 511 L/R (DLRP-rev1) pairs involved in autocrine/paracrine signaling, as previously described [11]. We considered 5 datasets, obtained with Affimetrix platforms, of 41 PTCs and the corresponding normal contralateral thyroid tissue samples (Table 1). Follicular thyroid carcinoma and all the other samples from different thyroid diseases were excluded for this analysis. When multiple probe sets or clones matching a single gene (either L or R) were present, each one was considered separately. Pearson's correlation coefficient was computed and a *P* value was assigned for each L/R pair in each dataset; the Bonferroni correction was applied because multiple tests were performed. Ligand and Receptor from pairs significantly correlated in at least 1 dataset were subsequently analyzed separately across the other datasets and were considered modulated when the gene expression of a partner of the pair was concordantly modulated at P<0.01 and/or FDR<0.25 in at least 3 datasets. TGFA and EGFR gene expression data of 51 PTCs, harboring known genetic mutations, and 5 normal thyroid tissues from pathologies other than thyroid cancer, reported in another publicly available dataset obtained on Affimetrix platform [9], were analyzed for possible correlations between expression levels and genetic mutations.

### Antibodies (Abs) and reagents

The following monoclonal Abs were used in blotting experiments: anti-phospho-tyrosine, clone 4G10, from Upstate Biotechnology (Lake Placid, NY); anti-MAP kinase activated (pERK1/2) from Sigma-Aldrich; and anti-phospho-AKT (Ser473), clone D9E, from Cell Signaling Technology. The following rabbit polyclonal Abs were used in blotting experiments: anti-phospho-EGFR (Tyr1173), anti-EGFR, and anti-Ret from Santa Cruz Biotechnology; anti-MAP kinase (ERK1/2) and anti-actin from Sigma-Aldrich; and anti-AKT from Cell Signaling Technology. The goat polyclonal Ab anti-EGFR from R&D Systems (Minneapolis, MN) was used in IP experiments. The following reagents were used in FACS experiments: the anti-EGFR monoclonal Ab MINT-5 (IgG1) [30] an affinity purified mouse IgG1 from eBioscience, as isotype control, and fluorochrome-conjugated secondary Ab (Becton Dickinson, Franklin Lakes, NJ). TGFA, and AG1478/Tyrphostin were from Sigma-Aldrich. The MEK inhibitor UO126 was from Promega and the PI3K inhibitor Ly294002 was from Calbiochem (La Jolla, CA); Erbitux® was used for Cetuximab. TaqMan® Gene Expression Assays were from Applied Biosystems (Foster City, CA), ELISA for TGFA dosage was from R&D Systems; and fluorochrome-conjugated Alexa Fluor 488 secondary Ab was from Molecular Probes (Invitrogen San Francisco, CA).

### Cell Culture

Five human thyroid cell lines were used in the present study: Normal immortalized N-Thy-ori3-1 [14] and PTC-derived NIM-1 [31], TPC1, B-CPAP [32], and K1 (European Collection of Cell Cultures, ECACC, Porton Down Salisbury, UK) cells. The mutational status of these cell lines are reported in Table S1. Genomic sequence on DNA from NIM-1 cells detected no mutation in EGFR gene (data not shown). Primary cultures of normal human thyroid cells infected with retroviral vectors containing RET/PTC1 oncogene were previously described [6]. HeLa cells (ATCC) were also used. All cell lines were maintained in DMEM with 10% fetal bovine serum (FBS), except for N-Thy-ori3-1 and K1 or primary thyrocytes, which were cultured in RPMI-1640 and in DMEM:HAM'S F12:MCDB 105 (2∶1∶1) supplemented with 10% FCS, respectively. The cells were maintained at 37°C in a humidified incubator under 5% carbon dioxide.

### Tumor Samples

Thyroid samples, 5 normal and 23 papillary thyroid carcinomas, were selected for this study (Table S2). The normal thyroid tissues were from patients with pathologies other than thyroid cancer. The samples were collected at the Department of Pathology at the Fondazione IRCCS Istituto Nazionale dei Tumori (Milan, Italy) from 1984 to date. Genetic lesions were characterized as previously described [8].

### RNA Extraction and real-time RT-PCR Analysis

Total RNA from tissue specimens was extracted essentially as described earlier [8]. For each sample, 20 ng of template was amplified in PCR reactions carried out in triplicate on an ABI PRISM 7900 using TaqMan® Gene Expression Assay (Applied Biosystems). EGFR, TGFA, and AREG were tested. PGK1 was used as housekeeping gene. Data analysis was performed using the SDS (Sequence Detection System) 2.2.2 software.

### FACS and IF analyses

Membrane evaluation of EGFR expression was performed as described earlier [33]. For IF analysis, cells grown on monolayer were fixed with 2% buffered paraformaldehyde and permeabilized with 0.2% Triton-X100. Incubations with primary and secondary Abs were performed essentially as described earlier [34]. The samples were analyzed using an Eclipse TE2000-S microscope with a 40X PanFluor objective (Nikon). Images were acquired with ACT-1 software (Nikon) at a resolution of 2250×1800 pixels.

### ELISA

The supernatants of the indicated cell lines, maintained in serum-free medium for 48 h, were analyzed. The amounts of TGFA released in the supernatant were quantified by ELISA with a commercial kit by R&D Systems. The results were normalized for cell density.

### Western blotting and IP

The total cell lysates, Western blotting, and IP were performed essentially as described earlier [35]. The blots were viewed and analyzed using ChemiDoc XRS and the Quantity One® software (Bio-Rad, Hercules, CA).

### Cell Proliferation

Cells were seeded at 20,000 cells/cm^2^ density in 96-well plates in the presence of DMEM with 10% FBS. The day after seeding, the cells were serum starved and exposed to solvent or different concentrations of drugs as indicated. Cell growth was evaluated by sulforhodamine B (SRB) colorimetric assay after 24, 48, and 72 h of treatment as previously described [36]. The experiment was performed in eight replicates.

### Statistical analysis

GraphPad Prism software (GraphPad Software, San Diego, CA) was used to analyze all the data. Significance of differences was determined by Student's *t*-test.

## Supporting Information

Table S1 Characteristics of the PTC cell lines used.
(0.03 MB DOC)

Table S2 Characteristics of the PTC tissue samples analyzed by real real-time RT-PCR.
(0.05 MB DOC)
