# Supplementary material for: Integrated Ligand-Receptor Bioinformatic and In Vitro Functional Analysis Identifies Active TGFA/EGFR Signaling Loop in Papillary Thyroid Carcinomas
Source: PLoS One. 2010 Sep 22;5(9):e12701. doi: 10.1371/journal.pone.0012701 (PMC2943897; doi:10.1371/journal.pone.0012701)
Supplement: Table S1 — Characteristics of the PTC cell lines used. (0.03 MB DOC) [file pone.0012701.s001.doc]

**Table 1S.**

| **CELL LINE** | **HISTOTYPE** | **GENETIC LESION** |
| --- | --- | --- |
| N-thy-ori3-1 | Immortalized Normal Thyrocytes (SV40) |  |
| TPC1 | PTC | RET/PTC1 |
| K1 | PTC | BRAF-V600E; PIK3CA-E542K |
| NIM-1 | PTC (metastasis to the sacral bone) | BRAF-V600E |
| B-CPAP | PTC | BRAF-V600E; p53-D259Y |
