# Supplementary material for: Integrated Ligand-Receptor Bioinformatic and In Vitro Functional Analysis Identifies Active TGFA/EGFR Signaling Loop in Papillary Thyroid Carcinomas
Source: PLoS One. 2010 Sep 22;5(9):e12701. doi: 10.1371/journal.pone.0012701 (PMC2943897; doi:10.1371/journal.pone.0012701)
Supplement: Table S2 — Characteristics of the PTC tissue samples analyzed by real real-time RT-PCR. (0.05 MB DOC) [file pone.0012701.s002.doc]

**Table 2S.**

| Sample no. | Genetic lesion | Primary tumor/ Lymphonodal metastasis | pT | pN |
| --- | --- | --- | --- | --- |
| 1 | BRAFV600E | T | 3 | 1 |
| 2 | BRAFV600E | T | 3 | 1 |
| 3 | BRAFV600E | T | 3 | 1 |
| 4 | BRAFV600E | M | 3 | 1b |
| 5 | BRAFV600E | T | 2 | 0 |
| 6 | BRAFV600E | T | 1b | x |
| 7 | BRAFV600E | T | 1a | 0 |
| 8 | RET/PTC3 | T | 4 | 1b |
| 9 | RET/PTC3 | M | 4 | 1b |
| 10 | RET/PTC3 | T | 3 | 1 |
| 11 | RET/PTC1 | M | 3 | 1b |
| 12 | RET/PTC | T | 3 | 1b |
| 13 | TRK | T | 2 | x |
| 14 | TRK | M | 3 | 1b |
| 15 | TRK | T | 4a | 1b |
| 16 | Unknown | M | 3 | 1b |
| 17 | Unknown | M | 4 | 1b |
| 18 | Unknown | T | 2 | 0 |
| 19 | Unknown | T | 3 | 1 |
| 20 | Unknown | T | 1 / 2 | 0 |
| 21 | Unknown | T | 2 | x |
| 22 | Unknown | T | 3 | x |
| 23 | Unknown | T | 3 | x |
